# Pericardiocentesis or surgical drainage: A national comparison of clinical outcomes and resource use

**DOI:** 10.1371/journal.pone.0267152

**Published:** 2022-04-28

**Authors:** Chelsea S. Pan, Russyan Mark Mabeza, Zachary Tran, Cory Lee, Joseph Hadaya, Yas Sanaiha, Peyman Benharash

**Affiliations:** Cardiovascular Outcomes Research Laboratories (CORELAB), David Geffen School of Medicine, University of California, Los Angeles, CA, United States of America; Shandong Public Health Clinical Center: Shandong Provincial Chest Hospital, CHINA

## Abstract

**Background:**

While institutional series have sought to define the optimal strategy for drainage of pericardial effusions, large-scale comparisons remain lacking. Using a nationally representative sample, the present study examined clinical and financial outcomes following pericardiocentesis (PC) and surgical drainage (SD) in patients admitted for pericardial effusion and tamponade.

**Methods:**

Adults undergoing PC or SD within 2 days of admission for non-surgically related pericardial effusion or tamponade were identified in the 2016–2019 Nationwide Readmissions Database. Multivariable logistic and linear models were developed to evaluate the association between intervention type and outcomes. The primary outcome of interest was mortality while secondary endpoints included reintervention, periprocedural complications, hospital length of stay (LOS), hospitalization costs and 30-day non-elective readmission.

**Results:**

Of an estimated 44,637 records meeting inclusion criteria, 28,862 (64.7%) underwent PC while the remainder underwent SD for initial management of pericardial effusion or tamponade. PC was associated with significantly increased odds of in-hospital mortality, reintervention and 30-day readmission relative to SD. PC was also associated with greater odds of cardiac complications but lower odds of infection, respiratory failure and blood transfusions compared to SD. Although PC was associated with shorter index hospital length of stay and costs, the two strategies yielded similar 30-day cumulative costs.

**Conclusion:**

Management of pericardial effusion with PC is associated with greater odds of mortality, reintervention and 30-day readmission but similar 30-day cumulative costs compared to SD. In the setting of adequate hospital capability and operator expertise, SD is a reasonable initial treatment strategy for pericardial effusion.

## Introduction

Pericardial effusion and tamponade are notable sequelae of several disease states and are increasingly diagnosed owing to advances in cardiovascular imaging [1, 2]. Definitive treatment of effusion relies on the removal of fluid either by pericardiocentesis or open surgical drainage. While both management strategies have been shown to be effective, current guidelines recommend that the intervention choice be guided by center and operator experience [3–5].

Limited clinical studies have reported similar acute mortality between pericardiocentesis and surgical drainage but differing rates of complications [6–10]. In a single-center study, Patel et al. found percutaneous management of malignancy related pericardial effusion to be associated with lower risk of complications while maintaining diagnostic value [6]. Specifically, they demonstrated patients undergoing pericardiocentesis were less likely to experience cardiac arrhythmias and prolonged mechanical intubation compared to surgical drainage [6]. Importantly, several other studies have demonstrated higher rates of fluid re-accumulation with this approach, occurring in as high as 30% of patients [7–10]. While institutional series have sought to define the optimal strategy for drainage of pericardial effusions, large-scale comparisons focusing on clinical and financial endpoints remain lacking.

The present study evaluated contemporary trends in the use of pericardiocentesis and surgical drainage. Using a nationally representative sample, we compared clinical and financial outcomes between the two drainage approaches in patients with non-surgically related pericardial effusions and tamponade. We hypothesized the two strategies would have similar risk of mortality and associated hospitalization costs but pericardiocentesis would carry greater risk of reintervention and readmission.

## Methods

### Data source and study population

This was a retrospective cohort study using the 2016–2019 Nationwide Readmissions Database (NRD). The NRD is the largest all-payer readmissions database and is part of the Healthcare Cost and Utilization Project (HCUP), a national effort focused on addressing healthcare outcomes and costs. Sampling from 28 participating states, the NRD provides estimates for approximately 58% of hospitalizations in the United States. Patients are followed across hospitalizations within each calendar year, allowing for tracking of readmissions.

All adult non-elective hospitalizations for pericardial effusion or tamponade were identified using relevant International Classification of Disease, Tenth Revision (ICD-10) diagnosis codes (S1 Table). Patients were classified as PC if they underwent pericardiocentesis and SD if they had surgical drainage within two days of index admission. Records with a concomitant diagnosis of traumatic injury or other cardiac operations were excluded to maintain homogeneity (6% excluded). Additionally, those missing key data or discharges in December of each year were excluded to allow for adequate follow up.

### Variable definitions and study outcomes

Patient and hospital characteristics were defined according to the NRD Data Dictionary and included age, sex and primary payer along with hospital location and teaching status [11]. Comorbidities were defined using ICD-10 codes and the Elixhauser comorbidity index, a validated composite score of 30 common conditions used to quantify the burden of chronic disease [12]. Hospitalization charges were calculated from cost-to-charge ratios provided by HCUP and adjusted for inflation using the 2019 Personal Consumption Expenditure Health Price Index [13].

The primary outcome of interest was mortality while secondary outcomes included reintervention, periprocedural complications, hospital length of stay (LOS), hospitalization costs and 30-day non-elective readmission. Reintervention was defined as the need for repeat PC or SD during index hospitalization. Periprocedural complications included blood transfusion, acute respiratory failure, infectious complications (incisional site infection and sepsis) as well as cardiac complications (ventricular fibrillation, ventricular tachycardia and cardiac arrest). Principal readmission diagnoses were identified using the Centers for Medicare and Medicaid Services Diagnosis Related Groups (DRGs) [14].

### Statistical analysis

All statistical analysis was performed with Stata 16.0 (StataCorp, LLC, College Station, TX, USA) using survey-specific methods to account for clustering at the hospital and regional level [11]. Trends were analyzed using a rank-based, non-parametric test developed by Cuzick (NP-trend) [15]. Continuous variables are reported as medians with interquartile range (IQR) and categorical variables are reported as frequencies. Mann-Whitney U and Pearson’s χ^2^ tests were used to compare continuous and categorical variables, respectively. Multivariable logistic and linear models were developed to evaluate the association between intervention type and outcomes. Elastic net regularization was used to select and minimize collinearity among the chosen clinically relevant covariates and to increase the out-of-sample reliability of our models [16]. The final model was optimized using the area under the receiver operating characteristics curve, when appropriate. Logistic model outputs are reported as adjusted odds ratios (AOR) with reference group (ref) and linear regression outputs are reported as beta coefficients (β), with corresponding 95% confidence intervals (95% CI) for both. Subgroup analyses were performed between the pericardial effusion and tamponade groups as well as between common etiologies of pericardial effusion. Statistical significance was defined at α<0.05. Due to the deidentified nature of the database, this study was deemed exempt from full review by the Institutional Review Board at the University of California, Los Angeles.

## Results

### Patient characteristics

**Fig 1:**
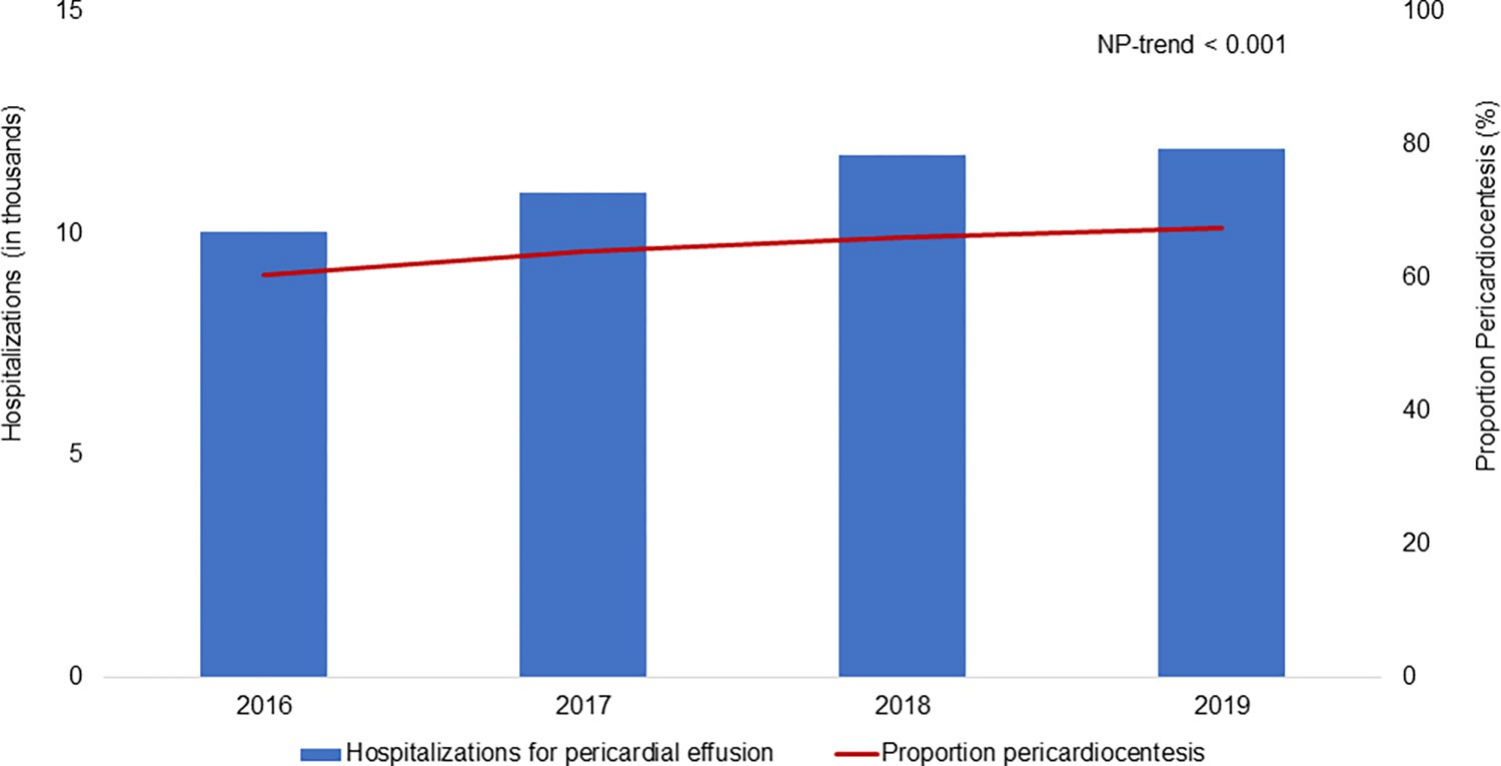
Trends of hospitalizations for pericardial effusion and pericardiocentesis utilization over study period. Abbreviations: *NP-trend*, Cusick’s non-parametric test for trend.

Of an estimated 44,637 records meeting inclusion criteria, 28,862 (64.7%) underwent pericardiocentesis (PC) while the remainder underwent surgical drainage (SD) for initial management of pericardial effusion or tamponade. As demonstrated in Fig 1, the proportion of patients initially managed with PC increased over the study period (2016: 60.3% to 2019: 67.5%, NP-trend <0.001). Compared to SD, the PC cohort was on average older (64 [53–74] vs 63 [52–73] years, p<0.001) but had a similar proportion of females (48.6 vs 48.8%, p = 0.83). Although the Elixhauser comorbidity index was similar between the two groups (4 [3–6] vs 4 [3–6], p = 0.40), the PC group had a significantly higher prevalence of heart failure, end-stage renal disease and liver disease, as demonstrated in Table 1. Furthermore, PC patients were more frequently diagnosed with tamponade relative to SD (68.0 vs 62.8%, p<0.001).

**Table 1:** Patient and hospital characteristics.

	PC	SD	*P*-Value
	n = 28,862	n = 15,775	
Age, years, median (IQR)	64 (53–74)	63 (52–73)	<0.001
Female sex, %	48.6	48.8	0.83
Elixhauser comorbidity index, median (IQR)	4 (3–6)	4 (3–6)	0.40
Comorbidities, %			
Arrhythmias	53.5	52.9	0.42
Autoimmune disease	21.6	21.1	0.43
Chronic lung disease	25.3	25.7	0.56
Congestive heart failure	32.6	30.5	0.002
Coronary artery disease	25.5	25.6	0.96
Diabetes	26.5	26.6	0.88
End-stage renal disease	9.72	7.81	<0.001
Hypertension	65.1	65.4	0.64
Liver disease	10.7	7.78	<0.001
Malignancy	37.3	39.0	0.02
Pericarditis	14.2	13.5	0.17
Peripheral vascular disease	10.0	10.1	0.85
Pulmonary circulation disorder	10.7	10.4	0.40
Valvular heart disease	13.2	16.5	<0.001
Indication, %			<0.001
Tamponade	68.0	62.8	
Effusion	32.0	37.2	
Etiology, %			<0.001
Neoplastic	36.0	37.7	
Inflammatory	12.8	12.4	
Renal	9.72	7.81	
Infectious	7.69	7.59	
Idiopathic	33.8	34.5	
Primary payer, %			<0.001
Medicare	54.1	51.5	
Private	28.8	30.4	
Medicaid	11.8	62.6	
Self-pay	5.29	6.45	
Hospital bed size, %			<0.001
Large	67.3	62.6	
Medium	23.2	27.3	
Small	9.57	10.1	
Hospital teaching status, %			<0.001
Metropolitan teaching	82.7	80.5	
Metropolitan non-teaching	13.9	17.9	
Non-metropolitan	3.37	1.57	

Abbreviations: *PC*, pericardiocentesis; *SD*, surgical drainage; *IQR*, interquartile range.

### Unadjusted outcomes

On unadjusted analysis, PC patients faced higher rates of in-hospital mortality compared to SD (10.2 vs 7.31%, p<0.001). Additionally, they more frequently necessitated reintervention (10.4 vs 0.81%, p<0.001). The majority of reinterventions were conversions from PC to SD (86.7%), while the remainder were PC to PC (9.24%), SD to PC (2.08%) or SD to SD (2.03%). In terms of periprocedural complications, PC patients more frequently experienced cardiac complications (8.64 vs 5.52%, p<0.001), but less frequently experienced infection (0.06 vs 0.30%, p<0.001), acute respiratory failure (1.59 vs 4.35%, p<0.001) or required blood transfusions (9.93 vs 11.7%, p<0.001). Compared to SD, the PC cohort had a shorter index LOS (5 [3–8] vs 6 [4–9] days, p<0.001) and incurred lower index hospitalization costs ($17,700 [$10,700-$30,300] vs $19,800 [$13,700-$31,200], p<0.001). However, there was no difference in 30-day cumulative costs ($23,900 [$13,300-$43,000] vs $25,400 [$15,700-$43,400], p = 0.56). Furthermore, despite shorter index LOS, PC patients were more likely to be readmitted within 30-days of discharge (21.6 vs 20.1%, p = 0.02). Relative to SD patients who were readmitted, PC patients were more frequently rehospitalized for cardiovascular related causes (41.2 vs 34.4%, p<0.001) while less commonly for pulmonary (12.6 vs 15.0%, p = 0.004) and infectious related causes (10.2 vs 13.4%, p<0.001).

### Risk-adjusted outcomes

**Fig 2:**
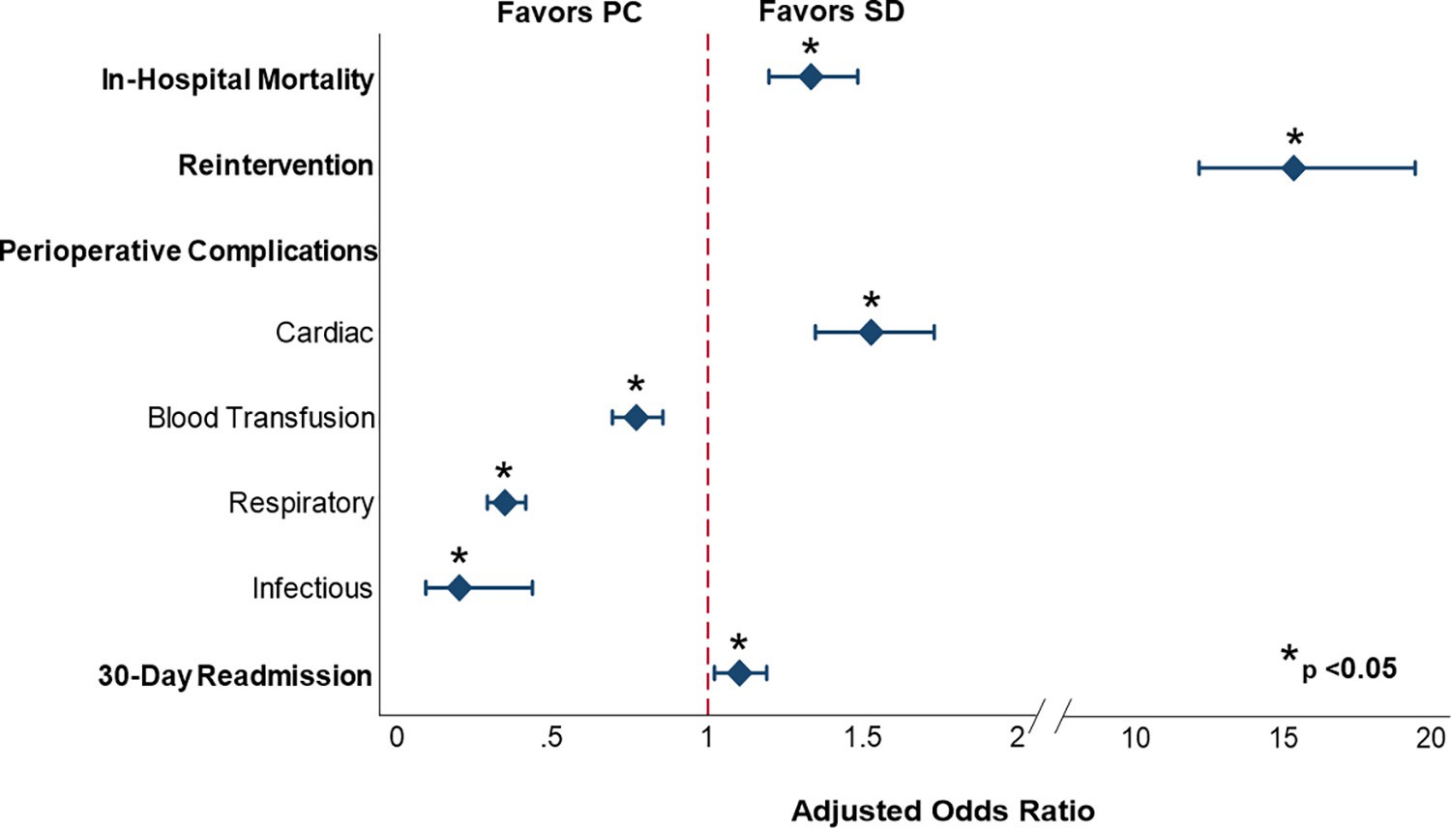
Independent association of pericardiocentesis with select outcomes of interest (Reference: Surgical drainage). Abbreviations: *PC*, pericardiocentesis; *SD*, surgical drainage.

After risk adjustment for patient and hospital characteristics, PC remained associated with significantly increased odds of in-hospital mortality relative to SD (AOR: 1.33, 95% CI: 1.20–1.48). Other factors associated with in-hospital mortality are detailed in S2 Table. PC was also associated with significantly greater odds of reintervention relative to SD (AOR: 14.6, 95% CI: 11.4–18.7). Additional factors associated with reintervention are listed in S2 Table. Regarding complications, the PC cohort was at greater risk for cardiac complications (AOR: 1.53, 95% CI: 1.35–1.73, ref: SD), but lower risk of infection (AOR: 0.20, 95% CI: 0.09–0.43, ref: SD), acute respiratory failure (AOR: 0.35, 95% CI: 0.29–0.41, ref: SD) and blood transfusions (AOR: 0.77, 95% CI: 0.69–0.86, ref: SD, Fig 2). On index hospitalization, PC remained associated with a shorter LOS (β: -1.12 days, 95% CI: -1.44 to -0.98) as well as lower hospitalization costs (β: -$4,280, 95% CI: -$5,400 to -$3,170) relative to SD (Table 2). However, this difference in cost did not persist upon aggregating 30-day cumulative costs (β: -$580, 95% CI: -$4,720 to $3,560), despite PC having greater odds of readmission (AOR: 1.10, 95% CI: 1.02–1.19, ref: SD). Subgroup analysis between the pericardial effusion and tamponade cohorts yielded similar adjusted outcomes (S3 Table). Additional results stratified by common etiologies of effusion are shown in S4 Table.

**Table 2:** Unadjusted and adjusted resource utilization stratified by treatment strategy (Reference: Surgical drainage).

	Unadjusted*			Adjusted^†^	
	PC	SD	*P*-value	AOR or β–coefficient	95% CI
	n = 28,862	n = 15,775			
**In-hospital mortality**	10.2	7. 31	<0.001	1.33	[1.20, 1.48]
**Reintervention**	10.4	0.81	<0.001	14.6	[11.4, 18.7]
**Complications**					
Cardiac	8.64	5.52	<0.001	1.53	[1.35, 1.73]
Infectious	0.06	0.30	<0.001	0.20	[0.09, 0.43]
Respiratory	1.59	4.35	<0.001	0.35	[0.29, 0.41]
Blood transfusion	9.93	11.7	<0.001	0.77	[0.69, 0.86]
**30-Day readmission**	21.6	20.1	0.02	1.10	[1.02, 1.19]
**Index hospitalization**					
LOS (days) (IQR)	5 (3–8)	6 (4–9)	<0.001	-1.21	[-1.44, -0.98]
Cost ($1,000) (IQR)	17.7 (10.7–30.3)	19.8 (13.7–31.2)	<0.001	-4.28	[-5.40, -3.17]
**30-Day cumulative**					
LOS (days) (IQR)	7 (4–13)	8 (5–14)	0.16	-0.77	[-1.76, 0.22]
Cost ($1,000) (IQR)	23.9 (13.3–43.0)	25.4 (15.7–43.4)	0.56	-0.58	[-4.72, 3.56]

Abbreviations: *PC*, pericardiocentesis; *SD*, surgical drainage; *AOR*, adjusted odds ratio; *95% CI*, 95% confidence interval; *LOS*, length of stay; *IQR*, interquartile range.

*Unadjusted outcomes reported as percentages or median with IQR.

†Adjusted outcomes reported as adjusted odds ratios or β-coefficient with corresponding 95% confidence intervals for both. SD as reference.

## Discussion

Pericardial effusion remains a significant cause for urgent hospitalization in the United States [2]. However, the relative merits of percutaneous and surgical drainage strategies continue to be debated [3–5]. Using a nationally representative cohort, we evaluated clinical and financial endpoints following percutaneous or open pericardial drainage. We found PC to be associated with significantly increased odds of in-hospital mortality, reintervention and 30-day readmission relative to SD. PC was also associated with greater odds of cardiac complications but lower odds of infection, respiratory failure and blood transfusions. Although PC was associated with shorter index hospital length of stay and costs, the two strategies yielded similar 30-day cumulative costs. Our findings build upon the body of literature examining the ideal management strategy for pericardial effusions and warrant further discussion.

Existing data comparing mortality after PC and SD are mixed. In studies examining the management of cardiac tamponade, both Allen et al. and Zgheib et al. found an approximate 4–5% increase in mortality following PC compared to SD [8, 17]. In contrast, several other studies found no difference in mortality between the two treatment strategies [7, 9]. The heterogeneity of findings from these largely single-institution studies may be explained, in part, by small sample sizes that may not be adequately powered to detect differences, as well as variation in hospital capability and provider experience. In the largest cohort study to date, we observed a 1.3-fold increase in relative odds of death in PC compared to SD. While selection bias in the choice of drainage strategy is undoubtedly a major factor, our results suggest the relative safety of surgical drainage in recent times. Nonetheless, careful consideration of patient history and clinical status remains critical in diagnostic and treatment decisions for managing pericardial effusions.

Congruent with previous studies, we observed that PC was associated with significantly greater odds of reintervention, a finding with implications on patient quality of life [7–10]. Our study builds on existing knowledge by detailing the various reintervention strategies utilized in patients with pericardial effusion. Of note, most reinterventions were conversions from PC to SD, suggesting that PC may often serve as a bridge to more definitive treatment with SD. Furthermore, we found PC was associated with lower odds of infectious, respiratory, and bleeding complications but higher odds of cardiac complications relative to SD. Compared to existing literature, our results are consistent regarding all complications except for cardiac [6]. Given the higher prevalence of cardiac tamponade in the PC cohort, our findings may reflect the higher level of acuity of patients undergoing PC.

In the current era of value-based medicine, there is a growing emphasis on delivering optimal care while minimizing healthcare-related costs. A recent study by Zgheib et al. found cardiac tamponade patients treated with SD to have lower mortality rates but longer length of stay and higher healthcare expenses [17]. Our study expands upon these findings in a larger, more contemporary cohort and includes long-term readmission outcomes. We found that although PC was associated with lower index hospitalization costs, this financial benefit did not persist when examining 30-day cumulative costs. It is possible that costs associated with readmission following PC outweigh its initial financial incentives. In light of the lower risk of mortality and reintervention as well as similar overall costs compared to PC, our results suggest that SD should be given more consideration as a definitive and cost-effective treatment option for pericardial effusion.

The present study has several important limitations. Due to the nature of the NRD as an administrative database, clinical data such as size of pericardial effusion, duration of drain placement, laboratory and imaging values were unable to be ascertained. Additionally, the etiology of the effusion was inferred from ICD-10 diagnosis codes but could not be directly verified with patient documentation. Clearly, the choice of drainage strategy was influenced by operator experience and decision making, resulting in potential bias that could not be accounted for. The database only includes information during the hospitalization period, and thus does not account for patients evaluated solely in the emergency department nor those experiencing out-of-hospital mortality. Despite these limitations, we used validated methodologies to report short- and medium-term outcomes for adults undergoing PC and SD.

In conclusion, management of pericardial effusion with PC is associated with greater odds of mortality, reintervention and 30-day readmission but similar 30-day cumulative costs compared to SD. In the setting of adequate hospital capability and operator expertise, SD is a reasonable initial treatment strategy for pericardial effusion.

## Supporting information

S1 Table International Classification of Diseases, Tenth Revision (ICD-10) diagnosis and procedures codes for identifying study cohort. (DOCX)

S2 Table Logistic model predicting in-hospital mortality and reintervention. Abbreviations: *AOR*, adjusted odds ratio; *95% CI*, 95% confidence interval; *Ref*, reference.
(DOCX)

S3 Table Adjusted outcomes stratified by pericardial effusion and tamponade (Reference: Surgical drainage). Abbreviations: *AOR*, adjusted odds ratio; *95% CI*, 95% confidence interval; *LOS*, length of stay.
(DOCX)

S4 Table Adjusted outcomes stratified by etiology of pericardial effusion (Reference: Surgical drainage). Abbreviations: *AOR*, adjusted odds ratio; *95% CI*, 95% confidence interval; *LOS*, length of stay.
(PPTX)
