# Supplementary material for: Pericardiocentesis or surgical drainage: A national comparison of clinical outcomes and resource use
Source: PLoS One. 2022 Apr 28;17(4):e0267152. doi: 10.1371/journal.pone.0267152 (PMC9049297; doi:10.1371/journal.pone.0267152)
Supplement: S1 Table — (DOCX) [file pone.0267152.s001.docx]

**Supplemental Table 1.** *International Classification of Diseases, Tenth Revision* (ICD-10) diagnosis and procedures codes for identifying study cohort

| **Diagnosis/Procedure** | **ICD-10 Code** |
| --- | --- |
| Pericardial effusion | I31.3 |
| Pericardial tamponade | I31.4 |
| Pericardiocentesis | 0W9D3ZX, 0W9D3ZZ, 02BN3ZX, 02BN3ZZ, 02NN3ZZ, 0W9D30Z, 02HN3YZ, 0W9D4ZX, 0W9D4ZZ, 02BN4ZX, 02BN4ZZ, 02NN4ZZ, 0W9D40Z, 02HN4YZ |
| Surgical drainage | 0W9D0ZX, 0W9D0ZZ, 02BN0ZX, 02BN0ZZ, 02NN0ZZ, 0W9D00Z, 02HN0YZ |
