# Supplementary material for: Pericardiocentesis or surgical drainage: A national comparison of clinical outcomes and resource use
Source: PLoS One. 2022 Apr 28;17(4):e0267152. doi: 10.1371/journal.pone.0267152 (PMC9049297; doi:10.1371/journal.pone.0267152)
Supplement: S2 Table — Abbreviations: AOR, adjusted odds ratio; 95% CI, 95% confidence interval; Ref, reference. (DOCX) [file pone.0267152.s002.docx]

**Supplemental Table 2**. Logistic model predicting in-hospital mortality and reintervention

|  | **Mortality** | | |  | **Reintervention** | | |
| --- | --- | --- | --- | --- | --- | --- | --- |
|  | **AOR** | ***P*-value** | **95% CI** |  | **AOR** | ***P*-value** | **95% CI** |
| Initial intervention |  |  |  |  |  |  |  |
| Surgical drainage | Ref |  |  |  | Ref |  |  |
| Pericardiocentesis | 1.33 | <0.001 | [1.20, 1.48] |  | 14.6 | <0.001 | [11.4, 18.7] |
|  |  |  |  |  |  |  |  |
| Demographics |  |  |  |  |  |  |  |
| Age, per year | 1.02 | <0.001 | [1.02, 1.02] |  | 0.99 | <0.001 | [0.98, 0.99] |
| Male | Ref |  |  |  | Ref |  |  |
| Female | 1.12 | 0.03 | [1.01, 1.25] |  | 0.95 | 0.34 | [0.85, 1.06] |
|  |  |  |  |  |  |  |  |
| Elixhauser comorbidity index, per unit | 1.30 | <0.001 | [1.26, 1.35] |  | 1.18 | <0.001 | [1.13, 1.22] |
|  |  |  |  |  |  |  |  |
| Comorbidities |  |  |  |  |  |  |  |
| Autoimmune disease | 0.56 | <0.001 | [0.48, 0.64] |  | 0.85 | 0.03 | [0.74, 0.98] |
| Chronic lung disease | 0.75 | <0.001 | [0.66, 0.84] |  | 0.94 | 0.37 | [0.81, 1.08] |
| Coagulopathy | 1.49 | <0.001 | [1.31, 1.69] |  | 1.06 | 0.50 | [0.90, 1.26] |
| Congestive heart failure | 0.83 | 0.003 | [0.73, 0.94] |  | 1.00 | 0.98 | [0.87, 1.15] |
| Coronary artery disease | 1.26 | <0.001 | [1.12, 1.41] |  | 0.89 | 0.11 | [0.78, 1.02] |
| Diabetes | 0.68 | <0.001 | [0.60, 0.77] |  | 0.93 | 0.34 | [0.81, 1.07] |
| End-stage renal disease | 1.22 | 0.02 | [1.04, 1.45] |  | 0.91 | 0.37 | [0.75, 1.11] |
| Hypertension | 0.47 | <0.001 | [0.42, 0.53] |  | 0.81 | 0.002 | [0.70, 0.93] |
| Liver disease | 1.74 | <0.001 | [1.51, 1.99] |  | 0.82 | 0.03 | [0.68, 0.99] |
| Malignancy | 0.96 | 0.49 | [0.86, 1.07] |  | 1.69 | <0.001 | [1.48, 1.94] |
| Pericarditis | 0.56 | <0.001 | [0.47, 0.66] |  | 1.54 | <0.001 | [1.32, 1.80] |
| Valvular heart disease | 0.52 | <0.001 | [0.44, 0.61] |  | 0.82 | 0.02 | [0.69, 0.97] |
|  |  |  |  |  |  |  |  |
| Primary payer |  |  |  |  |  |  |  |
| Private | Ref |  |  |  | Ref |  |  |
| Medicare | 1.12 | 0.08 | [0.97, 1.30] |  | 1.00 | 1.00 | [0.86, 1.16] |
| Medicaid | 1.19 | 0.11 | [1.00, 1.42] |  | 1.11 | 0.26 | [0.93, 1.32] |
| Self-pay | 1.44 | 0.16 | [1.16, 1.80] |  | 0.95 | 0.71 | [0.72, 1.25] |
|  |  |  |  |  |  |  |  |
| Indication |  |  |  |  |  |  |  |
| Effusion | Ref |  |  |  | Ref |  |  |
| Tamponade | 1.47 | 0.08 | [1.32, 1.64] |  | 1.44 | <0.001 | [1.25, 1.66] |
|  |  |  |  |  |  |  |  |
| Hospital teaching status | |  |  |  |  |  |  |
| Non-metropolitan | Ref |  |  |  | Ref |  |  |
| Metropolitan non-teaching | 0.89 | 0.46 | [0.66, 1.21] |  | 1.68 | 0.03 | [1.05, 2.68] |
| Metropolitan teaching | 0.77 | 0.08 | [0.58, 1.03] |  | 1.67 | 0.03 | [1.07, 2.63] |

Abbreviations: *AOR*, adjusted odds ratio; *95% CI*, 95% confidence interval; *Ref*, reference

C-statistic for mortality model: 0.73; C-statistic for reintervention model: 0.76
