# Supplementary material for: Pericardiocentesis or surgical drainage: A national comparison of clinical outcomes and resource use
Source: PLoS One. 2022 Apr 28;17(4):e0267152. doi: 10.1371/journal.pone.0267152 (PMC9049297; doi:10.1371/journal.pone.0267152)
Supplement: S3 Table — Abbreviations: AOR, adjusted odds ratio; 95% CI, 95% confidence interval; LOS, length of stay. (DOCX) [file pone.0267152.s003.docx]

**Supplemental Table 3.** Adjusted outcomes stratified by pericardial effusion and tamponade (Reference: Surgical drainage)

|  | **Pericardial effusion**  n=15,100 | | **Pericardial tamponade**  n=29,538 | |
| --- | --- | --- | --- | --- |
|  | **AOR or β−coefficient*** | **95% CI** | **AOR or β−coefficient*** | **95% CI** |
| **In-hospital mortality** | 1.49 | [1.22, 1.83] | 1.28 | [1.13, 1.46] |
|  |  |  |  |  |
| **Reintervention** | 11.4 | [7.43, 17.4] | 16.2 | [12.0, 21.8] |
|  |  |  |  |  |
| **Complications** |  |  |  |  |
| Cardiac | 1.58 | [1.25, 1.99] | 1.52 | [1.31, 1.75] |
| Infectious | 0.10 | [0.02, 0.56] | 0.23 | [0.10, 0.53] |
| Respiratory | 0.32 | [0.23, 0.46] | 0.35 | [0.29, 0.44] |
| Blood transfusion | 0.78 | [0.65, 0.94] | 0.77 | [0.68, 0.87] |
|  |  |  |  |  |
| **30-Day readmission** | 1.14 | [1.00, 1.30] | 1.08 | [0.99, 1.18] |
|  |  |  |  |  |
| **Index hospitalization** |  |  |  |  |
| LOS (days) | -1.51 | [-1.86, -1.16] | -1.04 | [-1.31, -0.77] |
| Cost ($1,000) | -5.14 | [-6.66, -3.61] | -3.73 | [-5.08, -2.39] |
|  |  |  |  |  |
| **30-day cumulative** |  |  |  |  |
| LOS (days) | -1.37 | [-2.73, -0.02] | -0.31 | [-1.41, 0.80] |
| Cost ($1,000) | -2.25 | [-9.02, 4.51] | 0.30 | [-4.79, 5.39] |

Abbreviations: *AOR*, adjusted odds ratio; *95% CI*, 95% confidence interval; *LOS*, length of stay

*Adjusted outcomes reported as adjusted odds ratios or β-coefficient with corresponding 95% confidence intervals for both. Surgical drainage as reference.
