# Supplementary material for: Pericardiocentesis or surgical drainage: A national comparison of clinical outcomes and resource use
Source: PLoS One. 2022 Apr 28;17(4):e0267152. doi: 10.1371/journal.pone.0267152 (PMC9049297; doi:10.1371/journal.pone.0267152)
Supplement: S4 Table — Abbreviations: AOR, adjusted odds ratio; 95% CI, 95% confidence interval; LOS, length of stay. (PPTX) [file pone.0267152.s004.pptx]

## Slide 1
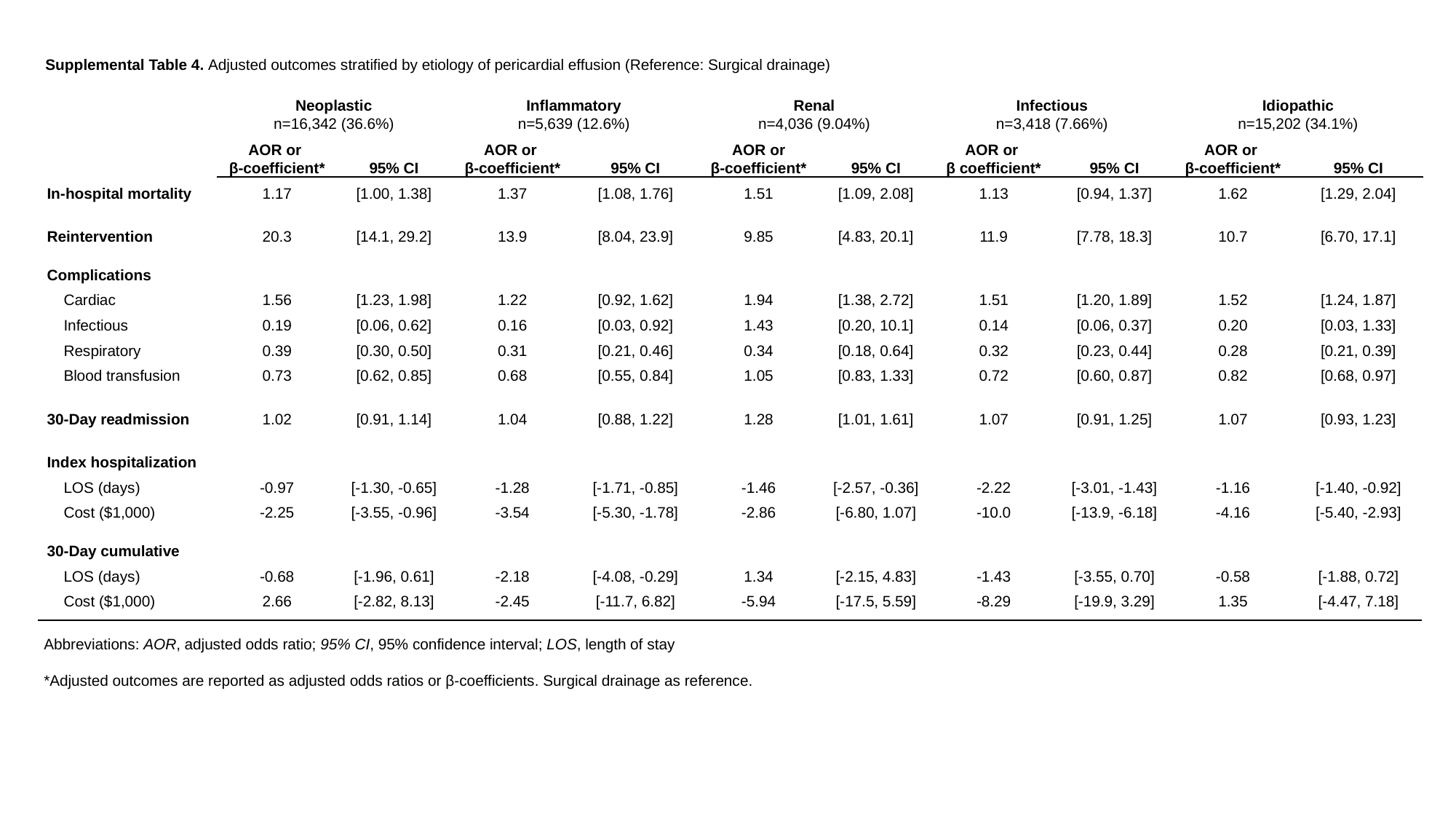

Supplemental Table 4. Adjusted outcomes stratified by etiology of pericardial effusion (Reference: Surgical drainage)
| | Neoplastic | | Inflammatory | | Renal | | Infectious | | Idiopathic | |
| --- | --- | --- | --- | --- | --- | --- | --- | --- | --- | --- |
| | n=16,342 (36.6%) | | n=5,639 (12.6%) | | n=4,036 (9.04%) | | n=3,418 (7.66%) | | n=15,202 (34.1%) | |
| | AOR or β-coefficient\* | 95% CI | AOR or β-coefficient\* | 95% CI | AOR or β-coefficient\* | 95% CI | AOR or β coefficient\* | 95% CI | AOR or β-coefficient\* | 95% CI |
| In-hospital mortality | 1.17 | [1.00, 1.38] | 1.37 | [1.08, 1.76] | 1.51 | [1.09, 2.08] | 1.13 | [0.94, 1.37] | 1.62 | [1.29, 2.04] |
| | | | | | | | | | | |
| Reintervention | 20.3 | [14.1, 29.2] | 13.9 | [8.04, 23.9] | 9.85 | [4.83, 20.1] | 11.9 | [7.78, 18.3] | 10.7 | [6.70, 17.1] |
| | | | | | | | | | | |
| Complications | | | | | | | | | | |
| Cardiac | 1.56 | [1.23, 1.98] | 1.22 | [0.92, 1.62] | 1.94 | [1.38, 2.72] | 1.51 | [1.20, 1.89] | 1.52 | [1.24, 1.87] |
| Infectious | 0.19 | [0.06, 0.62] | 0.16 | [0.03, 0.92] | 1.43 | [0.20, 10.1] | 0.14 | [0.06, 0.37] | 0.20 | [0.03, 1.33] |
| Respiratory | 0.39 | [0.30, 0.50] | 0.31 | [0.21, 0.46] | 0.34 | [0.18, 0.64] | 0.32 | [0.23, 0.44] | 0.28 | [0.21, 0.39] |
| Blood transfusion | 0.73 | [0.62, 0.85] | 0.68 | [0.55, 0.84] | 1.05 | [0.83, 1.33] | 0.72 | [0.60, 0.87] | 0.82 | [0.68, 0.97] |
| | | | | | | | | | | |
| 30-Day readmission | 1.02 | [0.91, 1.14] | 1.04 | [0.88, 1.22] | 1.28 | [1.01, 1.61] | 1.07 | [0.91, 1.25] | 1.07 | [0.93, 1.23] |
| | | | | | | | | | | |
| Index hospitalization | | | | | | | | | | |
| LOS (days) | -0.97 | [-1.30, -0.65] | -1.28 | [-1.71, -0.85] | -1.46 | [-2.57, -0.36] | -2.22 | [-3.01, -1.43] | -1.16 | [-1.40, -0.92] |
| Cost ($1,000) | -2.25 | [-3.55, -0.96] | -3.54 | [-5.30, -1.78] | -2.86 | [-6.80, 1.07] | -10.0 | [-13.9, -6.18] | -4.16 | [-5.40, -2.93] |
| | | | | | | | | | | |
| 30-Day cumulative | | | | | | | | | | |
| LOS (days) | -0.68 | [-1.96, 0.61] | -2.18 | [-4.08, -0.29] | 1.34 | [-2.15, 4.83] | -1.43 | [-3.55, 0.70] | -0.58 | [-1.88, 0.72] |
| Cost ($1,000) | 2.66 | [-2.82, 8.13] | -2.45 | [-11.7, 6.82] | -5.94 | [-17.5, 5.59] | -8.29 | [-19.9, 3.29] | 1.35 | [-4.47, 7.18] |
Abbreviations: AOR, adjusted odds ratio; 95% CI, 95% confidence interval; LOS, length of stay
*Adjusted outcomes are reported as adjusted odds ratios or β-coefficients. Surgical drainage as reference.
